# Supplementary material for: Factors associated with intention to be vaccinated with the COVID-19 booster dose: a cross-sectional study in Peru
Source: PeerJ. 2024 Mar 29;12:e16727. doi: 10.7717/peerj.16727 (PMC10984177; doi:10.7717/peerj.16727)
Supplement: Supplemental Information 1 [file peerj-12-16727-s001.docx]

**Supplementary Table 1:**

**Intention to be vaccinated with the booster dose according to type of survey of participants, Peru July 2022. (n=924)**

| Characteristic | | Intention to be vaccinated | | p |
| --- | --- | --- | --- | --- |
|  |  | No (n=110) | Yes (n=814) |  |
|  |  | n (%) | n (%) |  |
| Type of survey* | |  |  | 0.759 |
|  | Virtual | 100 (11.8) | 747 (88.2) |  |
|  | Physical | 10 (13.0) | 67 (87.0) |  |
|  |  |  |  |  |
| *Chi2 test was used. | | |  |  |
