# Supplementary material for: Factors associated with intention to be vaccinated with the COVID-19 booster dose: a cross-sectional study in Peru
Source: PeerJ. 2024 Mar 29;12:e16727. doi: 10.7717/peerj.16727 (PMC10984177; doi:10.7717/peerj.16727)
Supplement: Supplemental Information 3 [file peerj-12-16727-s003.docx]

**DATA COLLECTION SHEET**

**A. Generalities and Sociodemographic Factors:**

1. Age: _______ years
2. Sex:
   1. Male
   2. Female
3. Religion:
   1. Catholic
   2. Evangelical
   3. Atheist or agnostic
   4. Other religion
4. Marital status:
   1. Single
   2. Married
   3. Cohabitant
   4. Widower
   5. Divorced
5. What is the highest level of education you completed?
   1. Non-formal school
   2. Less than elementary school
   3. Complete elementary school
   4. Complete high school
   5. College, pre-university or full university
   6. Complete university graduate
6. Are you worried about having enough food for the next week?
   1. Very worried
   2. Somewhat worried
   3. Not very worried
   4. Not at all worried
7. The monthly economic income in your household is approximately:
   1. Greater than or equal to the minimum wage (≥1025 soles)
   2. Less than the minimum wage (<1025 soles)
8. In which region of Peru do you live?
   1. Coast
   2. Mountain range
   3. Jungle
9. Which of the following alternatives best describes the place where you currently live?
   1. Urban
   2. Rural
10. In which department of Peru do you reside?

_____________________

1. In the place where you normally live, do three or more people usually sleep in the same room?
   1. Yes
   2. No
2. How many children do you currently have?
   1. None
   2. One
   3. Two
   4. Three
   5. More tan three
3. Are you currently:
   1. Works
   2. Study
   3. Work and study
   4. Does not work and does not study
4. Are you related as a student or worker to health sciences careers such as human medicine, nursing, dentistry, obstetrics or psychology? (Answer this question only if you marked a, b or c in the previous one)
   1. Yes
   2. No

**B. Factors of perception of the booster dose against COVID-19:**

1. Do you agree to apply the booster dose against COVID-19? (Understand a booster dose as the third or fourth dose)
   1. Definitely yes
   2. Probably yes
   3. Probably not
   4. Definitely not
2. Do you think that the booster dose is effective and confers a protective effect against COVID-19?
   1. Definitely yes
   2. Probably yes
   3. Probably not
   4. Definitely not
3. How concerned are you about experiencing adverse effects from the booster dose? (Understand by booster dose as third or fourth dose)
   1. Very worried
   2. moderately concerned
   3. Little worried
   4. Nothing worried
4. How likely are you to accept the COVID-19 booster dose if it were recommended to you by friends and family?
   1. Most likely
   2. More or less the same
   3. Least likely
5. How likely are you to accept the COVID-19 booster dose if it were recommended to you by doctors and other health care providers?
   1. Most likely
   2. More or less the same
   3. Least likely
6. How likely are you to accept the COVID-19 booster dose if recommended by the World Health Organization (WHO)?
   1. Most likely
   2. More or less the same
   3. Least likely
7. How likely is it that you would accept the booster dose against COVID-19 if it were recommended to you by government health authorities in Peru? (For example: MINSA)
   1. Most likely
   2. More or less the same
   3. Least likely
8. How likely are you to accept the COVID-19 booster dose if it were recommended to you by politicians?
   1. Most likely
   2. More or less the same
   3. Least likely

**C. Clinical Factors:**

1. In the last 24 hours, have you had three or more of the following symptoms: fever, cough, shortness of breath, tiredness, muscle pain, sore throat, chest pain, nausea, loss of smell, eye pain or pain? upside down?
2. Yes
3. No
4. Are you currently pregnant? (Answer this question only if you put feminine in "sex")
5. Yes
6. No
7. During the last week, "I couldn't feel any positive feelings”.
8. It did not occur to me
9. Happened to me a little or part of the time
10. Happened to me quite a lot or for a good part of the time
11. Happened to me a lot or most of the time
12. During the last week, "It was difficult for me to take the initiative to do things."
13. It did not occur to me
14. Happened to me a little or part of the time
15. Happened to me quite a lot or for a good part of the time
16. Happened to me a lot or most of the time
17. During the last week, "I felt that there was nothing that excited me."
18. It did not occur to me
19. Happened to me a little or part of the time
20. Happened to me quite a lot or for a good part of the time
21. Happened to me a lot or most of the time
22. Durante la última semana, “Me sentí triste y deprimido”.
    1. It did not occur to me
    2. Happened to me a little or part of the time
    3. Happened to me quite a lot or for a good part of the time
    4. Happened to me a lot or most of the time
23. During the last week, "I felt sad and depressed."
    1. It did not occur to me
    2. Happened to me a little or part of the time
    3. Happened to me quite a lot or for a good part of the time
    4. Happened to me a lot or most of the time
24. During the last week, "I felt that I was worth very little as a person."
    1. It did not occur to me
    2. Happened to me a little or part of the time
    3. Happened to me quite a lot or for a good part of the time
    4. Happened to me a lot or most of the time
25. During the last week, "I felt that life had no meaning at all."
    1. It did not occur to me
    2. Happened to me a little or part of the time
    3. Happened to me quite a lot or for a good part of the time
    4. Happened to me a lot or most of the time
26. Have you ever had a heart condition such as myocarditis, pericarditis, deep vein thrombosis, myocardial infarction, or pulmonary embolism at any time in your life?
27. Yes
28. No
29. Have you ever had a respiratory condition such as respiratory failure, acute respiratory syndrome, pulmonary fibrosis, asthma or pneumonia at any time in your life?
30. Yes
31. No
32. I currently have:
33. 2 doses of vaccines against COVID-19
34. 3 doses of vaccines against COVID-19
35. 4 doses of vaccines against COVID-19
36. When any dose of COVID-19 vaccine was administered (either first, second, third or fourth dose) did you experience any adverse events?
37. Yes, mild, moderate (such as general malaise, headache, pain at the injection site, etc.)
38. Yes, severe (allergic reaction or requiring hospitalization)
39. No adverse events manifested
