# Supplementary material for: Factors associated with intention to be vaccinated with the COVID-19 booster dose: a cross-sectional study in Peru
Source: PeerJ. 2024 Mar 29;12:e16727. doi: 10.7717/peerj.16727 (PMC10984177; doi:10.7717/peerj.16727)
Supplement: Supplemental Information 4 [file peerj-12-16727-s004.docx]

**FICHA DE RECOLECCIÓN DE DATOS**

**A. Generalidades y Factores Sociodemográficos:**

1. Edad: _______años
2. Sexo:
   1. Masculino
   2. Femenino
3. Religión:
   1. Católica
   2. Evangélica
   3. Ateo o agnóstico
   4. Otra religión
4. Estado civil:
   1. Soltero
   2. Casado
   3. Conviviente
   4. Viudo
   5. Divorciado
5. ¿Cuál es el nivel de educación más alto que culminó?
   1. Escuela no formal
   2. Menos que escuela primaria
   3. Escuela primaria completa
   4. Escuela secundaria completa
   5. Facultad, preuniversidad o universidad completa
   6. Posgrado universitario completa
6. ¿Está preocupado por tener suficientes alimentos para la próxima semana?
   1. Muy preocupado
   2. Algo preocupado
   3. No muy preocupado
   4. Para nada preocupado
7. El ingreso económico mensual en su hogar es aproximadamente:
   1. Mayor o igual al sueldo mínimo (≥1025 soles)
   2. Menor al sueldo mínimo (<1025 soles)
8. ¿En qué región del Perú habita?
   1. Costa
   2. Sierra
   3. Selva
9. ¿Cuál de las siguientes alternativas describe mejor el lugar donde actualmente vive?
   1. Urbano
   2. Rural
10. ¿En qué departamento del Perú reside?

_____________________

1. ¿En el lugar donde normalmente habita, suelen dormir tres o más personas en un mismo cuarto?
   1. Sí
   2. No
2. ¿Cuántos hijos tiene actualmente?
   1. Ninguno
   2. Uno
   3. Dos
   4. Tres
   5. Más de tres
3. Actualmente usted:
   1. Trabaja
   2. Estudia
   3. Trabaja y Estudia
   4. No trabaja y no estudia
4. ¿Usted se encuentra relacionado como estudiante o trabajador a carreras de ciencias de la salud tales como medicina humana, enfermería, odontología, obstetricia o psicología? (Responder esta pregunta solo si en la anterior marcó a, b o c)
   1. Sí
   2. No

**B. Factores de percepción de la dosis de refuerzo contra la COVID-19:**

1. ¿Usted está de acuerdo en aplicarse la dosis de refuerzo contra la COVID-19? (Entiéndase por dosis de refuerzo como tercera o cuarta dosis)
   1. Definitivamente sí
   2. Probablemente sí
   3. Probablemente no
   4. Definitivamente no
2. ¿Cree que la dosis de refuerzo es eficaz y confiere un efecto protector contra la COVID-19?
   1. Definitivamente sí
   2. Probablemente sí
   3. Probablemente no
   4. Definitivamente no
3. ¿Qué tan preocupado se siente en experimentar efectos adversos por la dosis de refuerzo? (Entiéndase por dosis de refuerzo como tercera o cuarta dosis)
   1. Muy preocupado
   2. Moderadamente preocupado
   3. Poco preocupado
   4. Nada preocupado
4. ¿Qué tan probable es que usted acepte la dosis de refuerzo contra la COVID-19 si se lo recomendaran amigos y familiares?
   1. Más probable
   2. Más o menos, igual
   3. Menos probable
5. ¿Qué tan probable es que usted acepte la dosis de refuerzo contra la COVID-19 si se lo recomendaran médicos y otros profesionales de la salud que brindan atención médica?
   1. Más probable
   2. Más o menos, igual
   3. Menos probable
6. ¿Qué tan probable es que usted acepte la dosis de refuerzo contra la COVID-19 si se lo recomendara la Organización Mundial de la Salud (OMS)?
   1. Más probable
   2. Más o menos, igual
   3. Menos probable
7. ¿Qué tan probable es que usted acepte la dosis de refuerzo contra la COVID-19 si se lo recomendaran autoridades sanitarias gubernamentales del Perú? (Por ejemplo: MINSA)
   1. Más probable
   2. Más o menos, igual
   3. Menos probable
8. ¿Qué tan probable es que usted acepte la dosis de refuerzo contra la COVID-19 si se lo recomendaran políticos?
   1. Más probable
   2. Más o menos, igual
   3. Menos probable

**C. Factores Clínicos:**

1. ¿En las últimas 24 horas usted ha tenido tres o más de los siguientes síntomas: fiebre, tos, dificultad respiratoria, cansancio, dolor muscular, dolor de garganta, dolor de pecho, náuseas, pérdida del olfato, dolor de ojo o dolor de cabeza?
2. Sí
3. No
4. ¿Actualmente usted está gestando? (Responda esta pregunta solo si en “sexo” puso femenino)
5. Sí
6. No
7. Durante la última semana, “No podía sentir ningún sentimiento positivo”.
8. No me ocurrió
9. Me ocurrió un poco o durante parte del tiempo
10. Me ocurrió bastante o durante buena parte del tiempo
11. Me ocurrió mucho o durante la mayor parte del tiempo
12. Durante la última semana, “Se me hizo difícil tomar la iniciativa para hacer cosas”.
13. No me ocurrió
14. Me ocurrió un poco o durante parte del tiempo
15. Me ocurrió bastante o durante buena parte del tiempo
16. Me ocurrió mucho o durante la mayor parte del tiempo
17. Durante la última semana, “Sentí que no había nada que me ilusionara”.
18. No me ocurrió
19. Me ocurrió un poco o durante parte del tiempo
20. Me ocurrió bastante o durante buena parte del tiempo
21. Me ocurrió mucho o durante la mayor parte del tiempo
22. Durante la última semana, “Me sentí triste y deprimido”.
23. No me ocurrió
24. Me ocurrió un poco o durante parte del tiempo
25. Me ocurrió bastante o durante buena parte del tiempo
26. Me ocurrió mucho o durante la mayor parte del tiempo
27. Durante la última semana, “No me pude entusiasmar por nada.
28. No me ocurrió
29. Me ocurrió un poco o durante parte del tiempo
30. Me ocurrió bastante o durante buena parte del tiempo
31. Me ocurrió mucho o durante la mayor parte del tiempo
32. Durante la última semana, “Sentí que valía muy poco como persona”.
33. No me ocurrió
34. Me ocurrió un poco o durante parte del tiempo
35. Me ocurrió bastante o durante buena parte del tiempo
36. Me ocurrió mucho o durante la mayor parte del tiempo
37. Durante la última semana, “Sentí que la vida no tenía ningún sentido”.
38. No me ocurrió
39. Me ocurrió un poco o durante parte del tiempo
40. Me ocurrió bastante o durante buena parte del tiempo
41. Me ocurrió mucho o durante la mayor parte del tiempo
42. ¿Presentó en algún momento de su vida alguna afección cardíaca como miocarditis, pericarditis, trombosis venosa profunda, infarto de miocardio o embolia pulmonar?
43. Sí
44. No
45. ¿Presentó en algún momento de su vida alguna afección respiratoria como insuficiencia respiratoria, síndrome respiratorio agudo, fibrosis pulmonar, asma o neumonía?
46. Sí
47. No
48. Actualmente tengo:
49. 2 dosis de vacunas contra la COVID-19
50. 3 dosis de vacunas contra la COVID-19
51. 4 dosis de vacunas contra la COVID-19
52. Cuando se aplicó alguna dosis de la vacuna contra la COVID-19 (ya sea primera, segunda, tercera o cuarta dosis) ¿Presentó algún evento adverso?
53. Sí, leve, moderado (como malestar general, dolor de cabeza, dolor en la zona de inyección, etc.)
54. Sí, gravemente (Reacción alérgica o que requirió hospitalización)
55. No manifesté eventos adversos
